# Sapropterin dihydrochloride therapy in dihydropteridine reductase deficiency: Insight from the first case with molecular diagnosis in Brazil

**DOI:** 10.1002/jmd2.12224

**Published:** 2021-05-05

**Authors:** Charles Marques Lourenço, Janaina Dovidio, Isabela F. Lopes, Laís C. Silva, Marcela Almeida, Laura Vagnini, Jacqueline Fonseca, Zumira A. Carneiro, Beat Thöny

**Affiliations:** ^1^ Centro Universitário Estácio de Ribeirão Preto São Paulo Brazil; ^2^ Centro Paulista de Diagnóstico e Pesquisa em Genética Clínica São Paulo Brazil; ^3^ Laboratório DLE Bioquímica Genética Rio de Janeiro Brazil; ^4^ Division of Metabolism University Children's Hospital Zürich Switzerland

**Keywords:** BH_4_ deficiency, dihydropteridine reductase (DHPR) gene, hyperphenylalaninemia, l‐DOPA, tetrahydrobiopterin

## Abstract

Tetrahydrobiopterin (BH_4_) is a cofactor that participates in the biogenesis reactions of a variety of biomolecules, including l‐tyrosine, l‐3,4‐dihydroxyphenylalanine, 5‐hydroxytryptophan, nitric oxide, and glycerol. Dihydropteridine reductase (DHPR, EC 1.5.1.34) is an enzyme involved in the BH_4_ regeneration. DHPR deficiency (DHPRD) is an autosomal recessive disorder, leading to severe and progressive neurological manifestations, which cannot be exclusively controlled by l‐phenylalanine (l‐Phe) restricted diet. In fact, the supplementation of neurotransmitter precursors is more decisive in the disease management, and the administration of sapropterin dihydrochloride may also provide positive effects. From the best of our knowledge, there is limited information regarding DHPRD in the past 5 years in the literature. Here, we describe the medical journey of the first patient to have DHPRD confirmed by molecular diagnostic methods in Brazil. The patient presented with two pathogenic variants of the quinoid dihydropteridine reductase (*QDPR*) gene—which codes for the DHPR protein, one containing the in *trans* missense mutation c.515C>T (pPro172Leu) in exon 5 and the other containing the same type of mutation in the exon 7 (c.635T>C [p.Phe212Ser]). The authors discuss their experience with sapropterin dihydrochloride for the treatment of DHPRD in this case report.

**Synopsis**

DHPR deficiency leads to severe and progressive neurological manifestations, which can be controlled by l‐Phe‐restricted diets in association with the administration of dopaminergic agonists. Sapropterin dihydrochloride alone was able to prevent the neurodevelopmental delay progression in the absence of a l‐Phe‐restricted diet.

## 1 INTRODUCTION

Tetrahydrobiopterin (BH_4_) is a critical cofactor for several enzymes involved in the hydroxylation of aromatic amino acids, producing l‐tyrosine, l‐3,4‐dihydroxyphenylalanine (l‐DOPA), and 5‐hydroxytryptophan (5‐HTP). BH_4_ is also required for the production of nitric oxide and glycerol by different enzyme pathways. The synthesis and regeneration of BH_4_ is a multistage enzymatic process.^1^ The presence of pathogenic variants in the genes encoding these enzymes cause BH_4_ deficiencies, leading to a severe depletion of monoamine neurotransmitters (norepinephrine, epinephrine, dopamine, and indolamine serotonin), as well as hyperphenylalaninemia (HPA).^2^, ^3^, ^4^

Dihydropteridine reductase (DHPR, EC 1.6.99.7) is an enzyme involved in the regeneration of BH_4_. DHPR deficiency (DHPRD, OMIM 612676) is an autosomal recessive genetic disorder caused by mutations in the quinoid dihydropteridine reductase (*QDPR*) gene. This is a 7‐exon gene (NCBI numbers NG_008763.1 for genomic DNA and NM_000320.3 for cDNA), located in chromosome 4p.15.32, and it encodes the DHPR enzyme. Biallelic pathogenic variants in the *QDPR* gene lead to BH_4_‐deficient HPA and insufficient synthesis of monoamine neurotransmitters in the central nervous system (CNS).^1^

Patients with DHPRD commonly have an early symptoms onset. The symptoms include hypotonia or trunk hypotonia, movement disorders (mainly dystonia) with distal hypertonia, parkinsonism/hypokinetic rigid syndrome (consisting of bradykinesia or hypokinesia), extrapyramidal rigidity (“cogwheel rigidity”), rest tremor, impaired motor development and cognitive impairment, irritability and mood swings, neonatal dysphagia, lethargy, delayed language acquisition, temperature‐control disorders, and myoclonic seizures.^1^, ^5^, ^6^

The first documented case of DHPRD was in a 14‐year‐old male patient who had seizures and neurological deterioration even while being treated for a reputed phenylketonuria, which was diagnosed at the age of 3 weeks in 1975.^7^ Since then, researchers have cloned the human *QDPR* gene and reported a phenotypic diversity, thus allowing a greater understanding of the disease and treatment options. Overall, 303 cases had been listed in the BIODEF, an international database of BH_4_ deficiencies, as of October 2020. Besides this, the database contains 83 variants (unfortunately not giving details on the genotype). These mutations include missense and nonsense mutations, deletions, splice site alterations, and duplications or deletion‐insertions, and are spread over the 7 exons of the *QDPR* gene.

In accordance with the recent consensus guidelines for the diagnosis and treatment of BH_4_ deficiencies,^1^ all patients who presented with HPA in the newborn screening test should be referred to a specialised metabolic center for further diagnosis, prompt initiation of treatment, and appropriate follow‐up. The diagnosis of BH_4_ deficiencies includes (a) evaluation of blood l‐Phe concentration; (b) measurement of pterins in urine or blood samples; (c) lumbar puncture for the quantification of neurotransmitters and their metabolites in the cerebrospinal fluid (CSF); (d) evaluation of the enzymatic activity involved in the synthesis and regeneration of BH_4_. Genetic testing of the *QDPR* is also recommended to confirm the diagnosis.^4^, ^7^

The first‐line treatment of DHPR includes the administration of neurotransmitter precursors l‐DOPA/DOPA‐decarboxylase inhibitors and 5‐HTP in order to normalise the levels of monoamine neurotransmitters.^8^, ^9^ Treatment should be initiated as soon as possible and most likely continued throughout the patient's lifetime.^10^, ^11^ As cerebral folate deficiency is a major finding in DHPRD, folinic acid supplementation may also be required as soon as its levels are low. The administration of folinic acid (in combination with other medications) improves motor and cognitive functioning in patients with movement disorders or epileptic seizures.^1^ Dopamine agonists and monoamine oxidase inhibitors can also be considered as second‐line treatment in all BH_4_ deficiencies in combination with first‐line therapy if residual symptoms persist. Finally, in case of proven seizures, the administration of antiseizure drugs may be needed.

It has been shown that the HPA may be controlled with l‐Phe‐restricted diets and/or supplementation with sapropterin dihydrochloride (Kuvan, BioMarin Pharmaceuticals, Novato, California), a synthetic BH_4_ analogue. There is evidence that the restoration of BH_4_ bioavailability may prevent the neurodevelopmental delay in patients with BH_4_ deficiency.^12^, ^13^

In the present study, we report on the first case of a patient with DHPRD confirmed by molecular diagnosis in Brazil. Interestingly, the patient presented with a milder clinical phenotype of the disease, making the diagnosis more difficult. The therapeutic management, including the effects of sapropterin dihydrochloride, is documented here.

## 2 CASE REPORT

An 11‐year‐old male patient with young, non‐consanguineous parents was referred to a clinical geneticist to investigate hypotonia and persistent non‐phenylketonuria HPA.

Except for mild, premature placental displacement in the second month of pregnancy, no other complications occurred. There was no exposure to teratogenic agents during pregnancy. The patient was born at term by caesarean delivery in 2009. At birth, the patient's weight was 3115 g and his height was 52 cm. The APGAR evaluation was 10/10. Both the patient and mother were discharged 48 hours after the birth.

The neonatal screening test showed HPA (8.4 mg/dL, 509 μmol/L), which was confirmed in different samples. On the basis of this finding, the patient was diagnosed with phenylketonuria and was referred to an outpatient medical service for follow‐up treatment and a protein‐restricted diet was instituted.

The patient started walking at 1.3 years of age. Despite high adherence to a l‐Phe‐restricted diet, the patient's language acquisition was delayed (the first words were spoken at 2 years of age), resulting in referral to a speech therapist.

At 3 years of age, the patient had two seizure episodes accompanied by fever and cyanosis. Phenobarbital (2 mg/kg/day) was prescribed, and neuroimaging tests (computed tomography of the skull and magnetic resonance imaging of the brain) showed no evidence of abnormal activity. Electroencephalogram study showed diffuse disorganisation of the basal activity. The results were compatible with focal epilepsy.

The patient's caregivers denied that the patient had experienced any previous surgeries, other health issue, or prolonged hospitalization. No similar cases were found in the family. The caregivers stopped the l‐Phe‐restrictive diet when the patient was 3 years old because of the lack of cognitive improvement. Table 1 details the annual median of blood l‐Phe levels from 2009 to 2013. l‐Phe levels were consistently above the normal range (0.2‐4.0 mg/dL, 12‐240 μmol/L) during the evaluated period, revealing HPA.

**TABLE 1:** Five‐year evaluations of blood l‐Phe

Year of evaluation	Annual median ± SD (min; max)	Number of evaluations performed/year
2009	5.06 ± 6.5 (0.12; 16.32)	9
2010	1.36 ± 0.75 (0.6; 2.65)	6
2011^a^	1.61 ± 0.48 (0.94; 2.12)	7
2012	3.84 ± 4.27 (0.61; 13.25)	11
2013	4.23 ± 1.61 (2.8; 5.98)	3

*Note*: Tests were performed using a dried blood spot. Normal range was 0.2 to 4.0 mg/dL. SD: standard deviation; min and max: minimal and maximal obtained values, respectively.

^a^ Indicates the implementation of the dihydrochloride sapropterin treatment.

Due to the electroencephalographic alterations, the patient was referred to the Neurogenetics Service, where the hypothesis of a BH_4_ deficiency was formulated. Subsequently, the patient was subjected to an evaluation of neurotransmitters in the CSF and a DHPR activity assay, as detailed in Table 2. The diagnosis of DHPRD was confirmed by the low DHPR enzymatic activity together with the identification of two variants in the *QDPR* gene. More specifically, the genetic analysis was performed by PCR, followed by exon amplification of the *QDPR* gene, revealing that the patient is a compound heterozygote presenting two in *trans* missense‐mutated variants: c.515C>T (pPro172Leu) in exon 5 and c.635T>C (p.Phe212Ser) in exon 7. The first variant was inherited from the father and the second was inherited from the mother.

**TABLE 2:** Biochemical and genetic tests by the time of diagnosis

Assessment performed	Results
DHPR enzymatic activity	0.0 (NR: 1.8 to 3.8 mU/mg Hb)
Pterins in CSF	Neopterin: 20.1 (NR: 15‐35 nmol/L) Biopterin: 26 (NR: 20‐70 nmol/L) 5‐MTHF: 35.2 (NR: 64‐182 nmol/L)
Molecular study of *QDPR* gene	c.515C>T (p.Prol172Leu) in exon 5 c.635T>C (p.Phe212Ser) in exon 7

Abbreviations: CSF, cerebrospinal fluid; DHPR, dihydropteridine reductase; NR, normal range; QDPR, quinoid dihydropteridine reductase; and 5MTHF, 5‐methyltetrahydrofolic acid.

Physical assessment revealed symmetrical facies with mild facial diparesis, absence of ophthalmoplegia, unaltered eye movements, presence of mild tremors during rest, preserved muscle strength, mild dysmetria, dysdiadokinesia, generalized hypotonia, hypoactivity, cutaneous‐plantar osteotendinous reflexes in flexion, and atypical gait.

The initial treatment consisted of the association of l‐DOPA and carbidopa at the respective doses of 2 and 0.5 mg/kg and supplementation with 20 mg 5‐HT and 15 mg folinic acid. When l‐DOPA was administered three times per day, the patient failed to tolerate it and the drug was withdrawn. The l‐Phe‐restricted diet was reimplemented.

**FIGURE 1:**
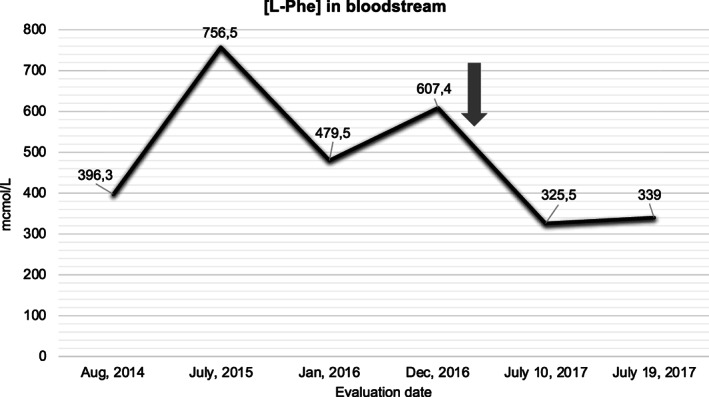
Three‐year blood l‐Phe evaluation. Blood l‐Phe tested from dried blood spot using tandem mass spectrometry (normal range: 25‐90.7 mcmol/L). The arrow represents the start of the 20 mg/kg/day sapropterin dihydrochloride treatment. The administration of 30 mg/kg/day was initiated in March 2019

In order to avoid neurodevelopmental delay, the administration of 20 mg/kg/day of sapropterin dihydrochloride started when the patient was 8 years old. Of note, sapropterin dihydrochloride remarkably reduced the blood l‐Phe levels of the patient, as shown in Figure 1. As the patient evolved with l‐DOPA‐induced dyskinetic movements, l‐DOPA was withdrawn and the dose of sapropterin dihydrochloride was increased to 30 mg/kg/day. Currently, this dose of sapropterin dihydrochloride has maintained the patient's l‐Phe blood levels within the normal range even with the absence of dietotherapy. The patient's blood level was 1.9 mg% in June 2020 (recommended range: 2‐6 mg%, dried blood spot evaluated by colorimetric enzyme assay).

## 3 DISCUSSION

In the case reported here, two distinct missense mutations inherited from each progenitor were found: c.635T>C (p.Phe212Ser) in exon 7 and c.515C>T (p.Prol172Leu) in exon 5. Notably, both mutations have previously been described as pathogenic.^14^ Considering heritability and genetic counseling, the chances of this couple having another child affected by this disease would be 25% in each pregnancy. In this particular patient, this genotype is associated with a milder phenotype, contributing to a delayed diagnosis.

The clinical presentation can be severe, specifically in contrast to other BH_4_Ds; however, the phenotypic spectrum is broad and mild cases are also described in DHPRD. In this case study, the patient did not present with severe manifestations in the first year of life. After that, the patient developed dysdiadochokinesia, generalized hypotonia, hypoactive osteotendinous reflexes, and difficulty in walking.

When HPA is detected in the newborn screening test, the evaluation of phenylalanine hydroxylase activity should be investigated in order to rule out phenylketonuria.^1^ In the event of HPA with unaltered activity of phenylalanine hydroxylase, dried blood spot, or urinary pterins dosage, DHPR enzyme activity must be performed, as well as lumbar puncture for the quantification of neurotransmitters and their metabolites in CSF. Genetic testing of the proband and the progenitors should also be performed; however, the testing is not already globally available. The CSF analysis can reveal reduced concentrations of the metabolites homovanillic acid and 5‐hydroxyindoleacetic acid, derived from the metabolism of dopamine and serotonin, respectively. Low levels of 5‐methyltetrahydrofolate (5‐MTHF) can also be detected.^15^ The investigation of pterins in CSF of the patient showed a reduced level of BH_4_, neopterin, and folates, which is compatible with DHPRD and this was confirmed by the molecular diagnosis. Moreover, as the clinical phenotype of BH_4_ deficiencies may overlap with numerous other disorders, such as cerebral palsy,^16^ early onset parkinsonism, oculogyric crises, and diurnal fluctuation of symptoms may be useful clinical features for differential diagnosis.^1^

The patient was treated with a combination of low doses of l‐DOPA and carbidopa, as well as 5‐HTP and folinic acid. It is worth noting that cases have been reported in which neonate patients with milder phenotypes were exclusively treated with restrictive diets.^17^ Although our patient adhered well to the diet, it was not sufficient to lower the blood l‐Phe concentration. Thus, to avoid neurological damage, the administration of monoamine neurotransmitter precursors was initiated. In spite of the treatment, the blood l‐Phe levels remained persistently increased and the patient continued to suffer seizures and neurodevelopmental delay. In fact, it has been reported that patients with DHPR may develop neurological symptoms even with blood l‐Phe levels within the normal range.^6^

Sapropterin dihydrochloride at the dose of 20 mg/kg/day was initiated to avoid neurodevelopmental delay. In parallel, the patient evolved with remarkable dyskinetic movements. However, as the patient could not tolerate the progressive staggering of the l‐DOPA doses, a combination of l‐DOPA and carbidopa was instituted. Since the administration of l‐DOPA per se can exacerbate the neurological symptoms,^4^ the dose of sapropterin dihydrochloride was increased to 30 mg/kg/day and the combination of l‐DOPA and carbidopa was withdrawn. Of note, this pharmacological treatment significantly reduced the dyskinetic movements and allowed the intake of natural protein in the diet. Finally, multidisciplinary monitoring with rehabilitation therapies, such as physical and occupational therapies, and psychopedagogy constituted a valuable strategy for the early detection and resolution of concerns related to the patient's disease.

## 4 CONCLUSIONS

We have described here the first Brazilian case of DPHRD confirmed by molecular diagnosis. In spite of a high adherence to a l‐Phe‐restricted diet, the patient progressed with neurodevelopmental delay (such as delayed language acquisition) and movement disorders, which were exacerbated by the staggering of the dopaminergic agonists. Sapropterin dihydrochloride, which was initially supplemented to prevent neurodevelopmental delay, was able to control HPA and the movement disorders, allowing the intake of natural proteins in the diet. This case report reinforces the need for investigation of BH_4_ deficiencies in all patients with increased blood l‐Phe concentrations who evolve with neurological symptoms.

## CONFLICT OF INTEREST

Charles M. Lourenço, Janaina Dovidio, Isabela F Lopes, Laís C Silva, Marcela Almeida, Laura Vagnini, Jacqueline Fonseca, Zumira A. Carneiro, and Beat Thöny declare no conflict of interest.
